# Transcriptional regulation of human osteopontin promoter by histone deacetylase inhibitor, trichostatin A in cervical cancer cells

**DOI:** 10.1186/1476-4598-9-178

**Published:** 2010-07-07

**Authors:** Priyanka Sharma, Santosh Kumar, Gopal C Kundu

**Affiliations:** 1 National Center for Cell Science, Pune, India

## Abstract

**Background:**

Trichostatin A (TSA), a potent inhibitor of histone deacetylases exhibits strong anti-tumor and growth inhibitory activities, but its mechanism(s) of action is not completely understood. Osteopontin (OPN) is a secreted glycoprotein which has long been associated with tumor metastasis. Elevated OPN expression in various metastatic cancer cells and the surrounding stromal cells often correlates with enhanced tumor formation and metastasis. To investigate the effects of TSA on OPN transcription, we analyzed a proximal segment of OPN promoter in cervical carcinoma cells.

**Results:**

In this paper, we for the first time report that TSA suppresses PMA-induced OPN gene expression in human cervical carcinoma cells and previously unidentified AP-1 transcription factor is involved in this event. Deletion and mutagenesis analyses of OPN promoter led to the characterization of a proximal sequence (-127 to -70) that contain AP-1 binding site. This was further confirmed by gel shift and chromatin immunoprecipitation (ChIP) assays. Western blot and reverse transcription-PCR analyses revealed that TSA suppresses c-jun recruitment to the OPN promoter by inhibiting c-jun levels while c-fos expression was unaffected. Silencing HDAC1 followed by stimulation with PMA resulted in significant decrease in OPN promoter activity suggesting that HDAC1 but not HDAC3 or HDAC4 was required for AP-1-mediated OPN transcription. TSA reduces the PMA-induced hyperacetylation of histones H3 and H4 and recruitment of RNA pol II and TFIIB, components of preinitiation complex to the OPN promoter. The PMA-induced expression of other AP-1 regulated genes like cyclin D1 and uPA was also altered by TSA. Interestingly, PMA promoted cervical tumor growth in mice xenograft model was significantly suppressed by TSA.

**Conclusions:**

In conclusion, these findings provide new insights into mechanisms underlying anticancer activity of TSA and blocking OPN expression at transcriptional level by TSA may act as novel therapeutic strategy for the management of cervical cancer.

## Background

Osteopontin (OPN) is a secreted, noncollagenous, sialic acid-rich, cytokine-like glycosylated phosphoprotein which is a member of **S**mall **I**ntegrin **B**inding **L****I**gand **N**-linked **G**lycoprotein (SIBLING) family and plays important role in determining the oncogenic potential of various cancers [1,2]. OPN expression is increased in a variety of cancers and is reported to correlate with enhanced tumor progression and metastasis [3-5]. OPN plays crucial roles in cancer cell metastasis, granuloma formation, dystrophic calcification and in coronary restenosis [6-11]. The prometastatic effects of OPN like cell adhesion, ECM invasion and cell proliferation are exhibited through interaction with its receptors which regulates various cell signaling pathways ultimately leading to tumor progression [12]. Earlier reports have shown that tumor-derived OPN is soluble and has close similarity with human milk OPN [13,14]. OPN is also found intracellularly, associated with ezrin and the cytoplasmic domain of CD44. It is important in transducing signals that leads to cytoskeletal rearrangements required during cell migration, fusion and bone resorption [1,15].

The expression of the genetic information encoded in the DNA is regulated largely by the chromatin structure [16]. Nucleosomes are the repeating units in chromatin which are composed of an octamer core of pairs of histones H2A, H2B, H3 and H4 wrapped with two superhelical turns of DNA around it [17]. Posttranslational covalent modifications of histones like acetylation and methylation of lysines and arginines and phosphorylation of serines have been shown to be important in gene regulation [18,19]. Acetylation of histones leads to neutralization of the positive charge of lysine residues, resulting in altered chromatin conformation. In this case, promoter regions of genes may be more accessible to transcription factor complexes [20].

The extent of histone acetylation is determined by the activities of the enzymes histone deacetylases (HDACs) and histone acetyltransferases (HATs) [21-24]. HAT or HDAC activity has been found to be disrupted in many cancers [25-29]. HDAC activity is increased in cancer cells and has been linked to carcinogenesis [30]. Various chemically and structurally diverse agents have been discovered which specifically inhibit HDAC activity. These include first natural product hydroxamate trichostatin A (TSA), suberoylanilide hydroxamic acid (SAHA), aliphatic compound valproic acid and several other natural and synthetic derivatives [31,32]. HDAC inhibitors exhibit growth inhibitory and anti-tumor activities like cell cycle arrest, inhibition of cell proliferation and induction of apoptosis in cancer cells both *in vitro *and tumor-bearing animals [33,34]. A number of HDAC inhibitors have been shown to be effective in phase I/II clinical trials. However, the mechanisms by which HDAC inhibitors regulate gene expression and show anti-tumor activities are not completely understood and remain to be elucidated [35].

In the present study, we have examined the effect of HDAC inhibitor, TSA on OPN transcription using human cervical carcinoma as a model. We found that TSA inhibits PMA-induced OPN gene expression. TSA suppressed the PMA-induced c-Jun recruitment to the OPN promoter by inhibiting c-Jun expression both at protein and RNA levels. Our results also identified the previously unrecognized AP-1 binding site in the human OPN promoter which is functionally active. We have also observed that TSA not only suppresses OPN transcription but also inhibits the expression of other AP-1 regulated genes like cyclin D1 and uPA. TSA suppresses the PMA-regulated tumor growth in mice xenograft model. In summary, these results suggest that HDAC inhibitor, TSA suppresses PMA-induced c-Jun expression leading to downregulation of AP-1 regulated genes like OPN, cyclin D1 and uPA. This data revealed that inhibiting OPN transcription by TSA might be an important therapeutic approach for the control of cervical cancer.

## Materials and methods

### Cell culture, antibodies and other reagents

The human cervical carcinoma cell lines, HeLa and SiHa were cultured in Minimum Essential Medium Eagle supplemented with 10% fetal bovine serum, 100 units/ml penicillin, 100 μg/ml streptomycin and 2 mM glutamine. The cells were maintained in a humidified atmosphere of 5% CO_2 _and 95% air at 37°C. The anti-OPN antibody was obtained from Chemicon International. The anti-HDAC4, anti-c-Jun, anti-c-Fos, anti-acetyl-H3, anti-acetyl-H4, anti-RNA pol II, anti-TFIIB, anti-cyclin D1, anti-uPA, anti-OPN and anti-actin antibodies were purchased from Santa Cruz Biotechnology. The anti-HDAC1 and anti-HDAC3 antibodies were from Upstate Biotechnology. PMA was purchased from Sigma. Trichostatin A (TSA) was obtained from Cell Signaling Technology. TSA was dissolved in minimum volume of ethanol, further diluted with media and used for *in vitro *and *in vivo *studies.

### Western blot analysis

HeLa cells were pretreated with TSA (0-1 μM) and then treated with PMA (50 ng/ml). In separate experiments, SiHa cells were treated with TSA (0-2 μM). Cells were lysed in lysis buffer and the lysates containing equal amount of total proteins (30-50 μg) were resolved by SDS-PAGE and blotted onto nitrocellulose membranes. The levels of OPN, c-Jun, c-Fos, cyclin D1 and uPA were analyzed by western blot using their specific antibodies.

### Electrophoretic Mobility Shift Assay (EMSA)

EMSA was performed as described previously [36]. HeLa cells were treated with PMA (50 ng/ml), nuclear extracts (5 μg each) were incubated with γ-^32^P-labeled double-stranded oligonucleotide containing the AP-1 binding site of OPN promoter in binding buffer (25 mM HEPES (pH 7.9), 0.5 mM EDTA, 0.5 mM DTT, 1% Nonidet P-40, 5% glycerol, and 50 mM NaCl) containing 2 μg of polydeoxyinosinic deoxycytidylic acid (poly dI-dC). The sequence of the probe used was 5' AAC CTC ATG ACA CAA TCT CTC 3'. The DNA-protein complex was resolved on 8% native polyacrylamide gel and analyzed by autoradiography. For supershift assay, the PMA-treated nuclear extracts were incubated with either anti-c-Jun or anti-c-Fos antibody and then EMSA was performed.

### RNA isolation and reverse transcription-PCR

RNA isolation and reverse transcription-PCR were performed as mentioned earlier [37]. Briefly, HeLa cells were pretreated with TSA (0-1 μM) followed by treatment with PMA (50 ng/ml). In separate experiments, SiHa cells were treated with TSA (0-2 μM). Total RNA was extracted using Trizol reagent (GIBCO BRL, Grand Island, NY) and reverse transcription-PCR was performed using following sets of primers. OPN (forward 5' AGA CCT GAC ATC CAG TAC CCT G 3', reverse 5' GTG GGT TTC AGC ACT CTG GT 3'), c-jun (forward 5' ATG GAG TCC CAG GAG CGG ATC AA 3', reverse 5' GTT TGC AAC TGC TGC GTT AG 3'), c-fos (forward 5' AAC CGG AGG AGG GAG CTG ACT GAT 3' reverse 5' GGG CCT GGA TGA TGC TGG GAA CA 3'), cyclin D1 (forward 5' CTT CCT CTC CAA AAT GCC AG 3', reverse 5' AGA GAT GGA AGG GGG AAA GA 3'), uPA (forward 5' CAC GCA AGG GGA GAT GAA 3', reverse 5' ACA GCA TTT TGG TGG TGA CTT 3') and actin (forward 5' GGC ATC CTC ACC CTG AAG TA 3', reverse 5' GGG GTG TTG AAG GTC TCA AA 3'). Aliquots of PCR products were analyzed by electrophoresis using 1.2% agarose gel.

### Chromatin immunoprecipitation (ChIP) assay

This assay was performed with ChIP assay kit (Upstate Biotechnology, Temecula, CA) according to the manufacturer's instructions. Briefly, cells were cross-linked in 1% formaldehyde solution for 10 min at room temperature. Cells were lysed in 300 μl of SDS lysis buffer and sonicated to generate 200-1000 bp DNA fragments. The cross-linked chromatin in sonicated fractions were immunoprecipitated with anti-p-c-Jun, anti-acetyl H3, anti-acetyl H4, anti-RNA pol II or anti-TFIIB antibody. DNA fragments were analyzed by PCR using specific primers which include AP-1 binding site of OPN promoter (forward 5'-TCT TCC TGG ATG CTG AAT GC-3', reverse 5'-CCA AGC CCT CCC AGA ATT TAA-3').

### Construction of expression vectors and site directed mutagenesis

The human OPN promoter fragments (-500/+20, -267/+20, -127/+20) cloned in pGL3 vector were generous gift from Dr. J. H. Chen (Tzu Chi University, Taiwan). Two deletion constructs of the promoter, -70/+20 and -20/+20 were generated by PCR with two 5' primers and a fixed 3' primer. The PCR amplified fragments were digested with *Kpn *I and *Sac *I and further cloned into promoter-less luciferase reporter plasmid pGL3-Basic (Promega, Medison, WI). Three mutation constructs namely CM, AM and OM were generated using QuickChange site-directed mutagenesis kit according to the manufacturer's instructions (Stratagene, La Jolla, CA) in which binding sites of CEBPα/AML-1, AP-1 and Oct-1/Oct-2 transcription factors are respectively mutated.

### Transfection and luciferase reporter assay

HeLa cells were seeded in 12-well plates and transfected with 2 μg of human OPN promoter deletion and mutation constructs using 4 μl of Lipofectamine 2000 as per the manufacturer's instructions. After 6 h of incubation, the medium was replaced with complete medium. The luciferase activity was measured in cell lysates using dual luciferase reporter assay kit from Promega and normalized to Renilla luciferase activity. Fold-changes in luciferase activity with respect to control were calculated.

### RNA interference

Cells were transiently transfected with HDAC1, HDAC3 (siRNA kit from Upstate Biotechnology, Temecula, CA) and HDAC4 (Santa Cruz Biotechnology, Santa Cruz, CA) specific siRNA using Lipofectamine 2000 according to the manufacturer's instructions. These siRNA transfected cells were used for reporter assay and western blot analysis.

### In vivo tumorigenicity, histopathology and immunofluorescence

The tumorigenicity and immunofluorescence experiments were performed as described [38]. Briefly, HeLa cells (1 × 10^6^/0.2 ml) were mixed with matrigel (1:1 ratio, BD Biosciences) and injected subcutaneously into the flanks of female NOD/SCID mice (6-8 weeks old). The mice were maintained under specific pathogen-free conditions according to the guidelines of Experimental Animal Facility (EAF) of National Centre for Cell Science. Six mice were used in each group of animals. PMA (10 μg/kg body weight) was injected intratumorally. In some experiments, two doses of TSA (0.5 mg/kg and 4 mg/kg body weight) along with PMA were injected to the site of the tumors. Tumor volume was estimated using the following formula: π/6 [(d1 × d2)^3/2^], where d1 and d2 are the two perpendicular diameters. Mice were sacrificed after 4 weeks and tumors were excised. The tumors were immediately snap-frozen in liquid nitrogen and the levels of OPN, c-Jun, uPA and cyclin D1 in the tumor lysates were analyzed by western blotting and reverse transcription-PCR. The remaining portions of the tumor samples were formalin fixed and tumor sections were analyzed by immunofluorescence using their specific antibodies.

### Statistical analysis

The results of luciferase reporter and tumorigenicity assay are expressed as mean ± SD and ± SEM respectively. Statistical differences were analyzed by Student's *t *test. A *P *value of < 0.05 was considered as significant. All bands were analyzed densitometrically and the fold changes were calculated (Kodak Digital Science).

## Results

### TSA suppresses PMA-induced OPN transcription in HeLa cells

**Figure 1:**
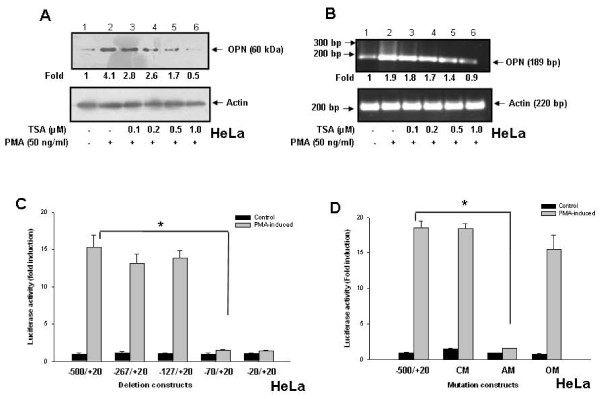
**TSA suppresses PMA-induced OPN transcription in HeLa cells**. **A**. HeLa cells were preincubated with 0-1 μM TSA for 1 h followed by treatment with PMA (50 ng/ml) for 6 h. Whole cell lysates were analyzed by western blot using anti-OPN antibody. **B**. HeLa cells were pretreated with TSA followed by PMA under similar conditions as described above. Total RNA was isolated and OPN mRNA levels were detected by semiquantitative RT-PCR and analyzed by agarose gel electrophoresis. Actin was used as control. The data represents three experiments exhibiting similar results. **C**. HeLa cells were cotransfected with hOPN promoter deletion constructs (-500/+20, -267/+20, -127/+20, -70/+20 and -20/+20) containing luciferase reporter gene along with renilla luciferase vector, pRL followed by stimulation with PMA. The luciferase activity was measured in cell lysates and normalized to Renilla luciferase activity. Fold-changes in luciferase activity with respect to control were calculated. Columns, mean of triplicate determinations; bar, S.D. *, p < 0.05. **D**. HeLa cells were cotransfected with various hOPN promoter mutation constructs (-500/+20, CM, AM and OM) containing luciferase gene along with pRL vector and treated with PMA. The luciferase activity was measured and normalized. Fold-changes in luciferase activity with respect to control were calculated. Columns, mean of triplicate determinations; bar, S.D. *, p < 0.05.

Earlier reports have shown that stimulation of smooth muscle cells with PMA results in induction of OPN gene expression [11]. To determine whether HDAC inhibitor, TSA could suppress PMA-induced OPN expression, HeLa cells were pretreated with TSA (0-1 μM) for 1 h before being treated with PMA. Expression of OPN both at protein and mRNA levels was analyzed by western blot and reverse transcription-PCR respectively. It was observed that treatment of cells with TSA resulted in decrease in PMA-induced OPN expression in a dose dependent manner. The results showed that TSA was able to inhibit OPN expression both at protein (Figure 1A) as well as mRNA (Figure 1B) levels. To investigate whether TSA regulates OPN expression in other cervical cancer cells, highly invasive SiHa cells were treated with TSA. The data revealed that TSA suppresses OPN transcription at protein and mRNA levels in these cells also (Additional file 1, Figure S1A and Figure S1B).

### Deletion and mutagenesis analyses of OPN promoter

The proximal segment of human OPN promoter and its sequence containing various transcription factor binding sites are shown (Additional file 2, Figure S2A and Figure S2B). To characterize the regulatory sequence involved in PMA-induced activation of OPN transcription, a proximal segment (-500 to +20 region) in the human OPN promoter containing C/EBPα/AML-1, AP-1 and Oct-1/Oct-2 transcription factor binding sites was taken into consideration. A region from -500 to +20 of the hOPN promoter sequence and serially deleted promoter sequences (-267/+20, -127/+20, -70/+20 and -20/+20) were cloned into promoter-less luciferase reporter plasmid, pGL3-Basic. The luciferase activity was analyzed in HeLa cells cotransfected with OPN promoter constructs along with Renilla. When luciferase activities of transfected cells were assayed, deletion of the region between -127 and -70 resulted in a dramatic decrease in OPN promoter activity suggesting that AP-1 binding site located in this region might be responsible for OPN transcription in response to PMA (Figure 1C). To further confirm the role of AP-1 binding sequence in PMA-induced OPN expression, mutation constructs were generated, namely CM, AM and OM in which C/EBPα/AML-1, AP-1 and Oct-1/Oct-2 binding sites were respectively mutated. Mutations within the AP-1 binding sequence caused significant reduction in the promoter activity upon PMA treatment (Figure 1D) suggesting the involvement of AP-1 in PMA-induced OPN transcription.

### Identification of transcription factor involved in PMA-induced OPN expression

**Figure 2:**
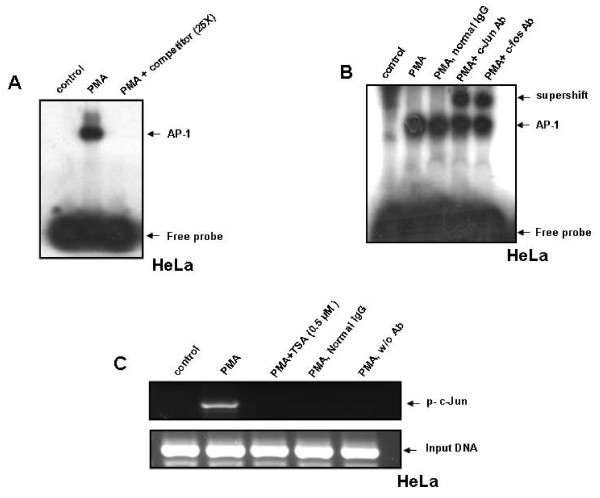
**PMA induces AP-1 DNA binding to the OPN promoter in HeLa cells**. **A**. HeLa cells were treated with PMA (50 ng/ml) for 2 h. Nuclear extracts were prepared and incubated with ^32^P-labeled probe containing AP-1 binding site of OPN promoter and analyzed by EMSA. **B**. For supershift assay, nuclear extracts from PMA-treated HeLa cells were incubated with anti-c-Jun or anti-c-Fos antibody and then analyzed by EMSA. **C**. HeLa cells were pretreated with TSA (0.5 μM) for 1 h and then with PMA (50 ng/ml) for 2 h. Cross-linked chromatin fragments were immunoprecipitated with anti-p-c-Jun antibody and was PCR amplified using specific primers derived from the region of OPN promoter containing AP-1 binding site. For negative controls, normal mouse IgG was used or specific antibody was omitted.

The transcription factor binding to the OPN promoter in response to PMA was identified by electrophoretic mobility shift assay. The nuclear extract prepared from HeLa cells was incubated with labeled oligonucleotide probe containing AP-1 binding sequence of OPN promoter. A specific complex was detected in the nuclear extracts of PMA-treated HeLa cells and the unlabeled oligonucleotide could competitively inhibit the complex formation (Figure 2A) indicating the specificity of the DNA-protein interaction. Supershift assay was performed to investigate whether c-Jun or c-Fos could bind to the sequence in the probe using their specific antibodies. Addition of either of the antibody resulted in supershift indicating the presence of AP-1 heterodimer at the OPN promoter in response to PMA (Figure 2B). To confirm the *in vivo *recruitment of c-Jun to the OPN promoter, ChIP assay was performed. This assay reflects the binding of the protein to the endogenous gene. Cross-linked chromatin was immunoprecipitated using anti-p-c-Jun antibody, the reverse cross-linked DNA was PCR amplified using a set of primers containing AP-1 binding site of OPN promoter. The results showed the induction of recruitment of c-Jun to the OPN promoter upon PMA treatment which was inhibited by TSA (Figure 2C).

### TSA suppresses PMA-induced c-Jun but not c-Fos expression

**Figure 3:**
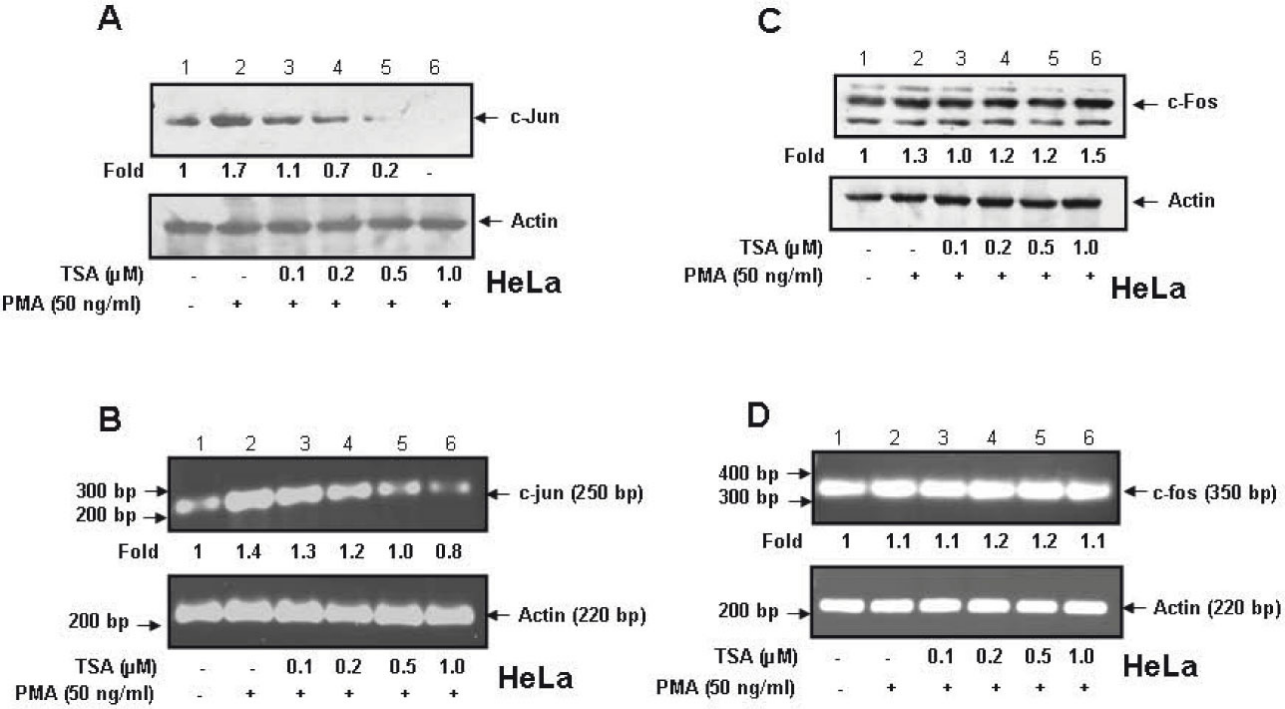
**TSA blocks PMA-induced c-Jun but not c-Fos expression in HeLa cells**. **A and C**. HeLa cells were pretreated with TSA (0-1 μM) for 1 h followed by treatment with PMA (50 ng/ml) for 2 h. Cell lysates (50 μg) containing equal amount of total proteins were analyzed by western blot using either anti-c-Jun or anti-c-Fos antibody. **B and D**. HeLa cells were pretreated with TSA and then with PMA under similar conditions as described above. Total RNA was isolated and the levels of c-jun and c-fos mRNAs were detected by semiquantitative RT-PCR. Actin was used as control.

One of the mechanisms by which recruitment of c-Jun to the OPN promoter could be affected is by alteration in its expression. To examine this hypothesis, c-Jun expression both at RNA and protein levels was detected in lysates of HeLa cells preincubated with TSA followed by treatment with PMA. The results showed that TSA suppresses PMA-induced c-Jun expression at protein (Figure 3A) as well as mRNA (Figure 3B) levels. In contrast, TSA did not have any effect on c-Fos expression (Figure 3C and Figure 3D) suggesting that the recruitment of c-Jun but not c-Fos is regulated by TSA. To further investigate whether this phenomenon is applicable to other cervical cancer cells, highly invasive SiHa cells were treated with TSA and the levels of c-Jun and c-Fos were analyzed. The data revealed that TSA suppresses c-Jun but not c-Fos expression both at protein and mRNA levels (Additional file 3, Figure S3 A-D).

### Determination of acetylation status of histones H3 and H4 in response to PMA and TSA

**Figure 4:**
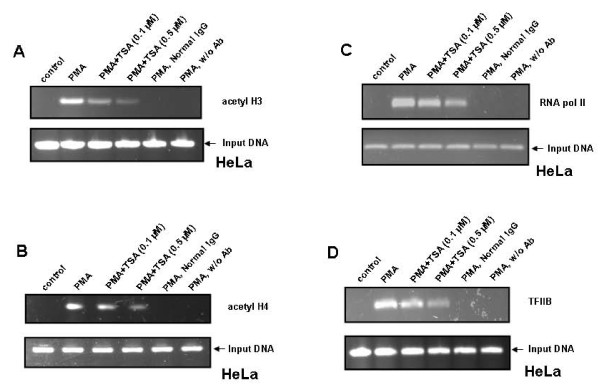
**TSA inhibits PMA-induced hyperacetylation of histones H3 and H4 and recruitment of RNA pol II and TFIIB to OPN promoter in HeLa cells**. **A-D**. HeLa cells were pretreated with TSA for 1 h and then with PMA for 2 h. Cross-linked DNA-protein complexes were immunoprecipitated with anti-acetyl H3, anti-acetyl H4, anti-RNA pol II or anti-TFIIB antibody and PCR amplified using specific primers derived from the region of OPN promoter containing AP-1 binding site. For negative controls, normal mouse IgG was used. The data represents three experiments exhibiting similar results.

Previous studies have demonstrated that HDAC inhibitors induce the accumulation of acetylated histones in both transformed and normal cells [39-41]. To investigate whether treatment of cells with PMA and TSA has any effect on the pattern of acetylation of histones associated with OPN promoter, we performed chromatin immunoprecipitation assays using anti-acetyl H3 and anti-acetyl H4 antibodies and primers containing the AP-1 binding site of OPN promoter. We found that exposure of cells with PMA led to hyperacetylation of histones H3 and H4 in the proximal region of OPN promoter. This PMA-induced hyperacetylation of histones was inhibited by TSA in a dose dependent manner (Figure 4A and Figure 4B).

### PMA enhances the recruitment of RNA pol II and TFIIB to OPN promoter, an effect that is reversed by TSA

Previous reports have shown that HDAC inhibitors interfere with the initiation of transcription by blocking the recruitment of components of pre-initiation complex [42]. Therefore, we next examined whether PMA and TSA have any effect on the association of RNA polymerase II and TFIIB with OPN promoter sequence having AP-1 binding site. Employing ChIP assays, we observed that stimulation of HeLa cells with PMA caused induction in the binding of RNA pol II and TFIIB to AP-1 binding region of OPN promoter. The results also revealed that TSA was able to abrogate the PMA-induced RNA pol II and TFIIB association with OPN promoter (Figure 4C and Figure 4D) in a similar manner as observed in the case of histones H3 and H4. These findings suggest that TSA altered the recruitment of components of basal transcription machinery.

### Silencing HDAC1 expression leads to decrease in PMA-induced OPN promoter activity

**Figure 5:**
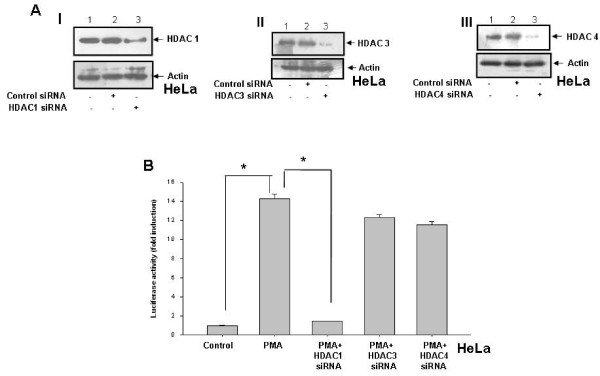
**HDAC1 is involved in regulation of PMA-induced OPN promoter activity in HeLa cells**. **A. panels I-III**. HeLa cells were transfected with either siRNAs to HDAC1, HDAC3 and HDAC4 or control siRNA. The expressions of various HDACs were analyzed by western blot using their specific antibodies. Actin served as loading control. **B**. HeLa cells were transfected with siRNAs to HDAC1, HDAC3 and HDAC4. These transfected cells were cotransfected with hOPN promoter (-500/+20) containing luciferase reporter gene along with Renilla luciferase, pRL vector and then treated with PMA. The luciferase activity was measured in cell lysates and normalized to Renilla luciferase activity. Fold-changes in luciferase activity with respect to control were calculated. Columns, mean of triplicate determinations; bar, S.D. *, p < 0.05.

TSA inhibits various histone deacetylases and exhibits little or no preference for HDACs [23,43]. Therefore, it is of great interest to characterize the class of HDAC involved in OPN transcription in response to PMA. The expressions of HDACs in lysates of siRNA transfected cells were analyzed by western blot to check the specificity of the siRNAs (Figure 5, panel A, I-III). The luciferase assay showed that HDAC1 siRNA but not HDAC3 or HDAC4 siRNA significantly decreased the PMA-induced OPN promoter activity (Figure 5, panel B) suggesting that HDAC1 is involved in PMA-induced OPN transcription.

### Expression of AP-1 regulated genes in response to PMA and TSA

**Figure 6:**
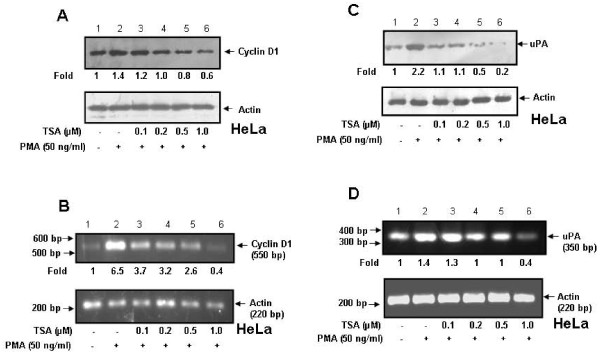
**Effect of TSA on cyclin D1 and uPA expression at protein and RNA levels in HeLa cells**. **A and C**. HeLa cells were pretreated with TSA (0-1 μM) for 1 h followed by treatment with PMA (50 ng/ml) for 6 h. Cell lysates were analyzed by western blot using anti-cyclin D1 or anti-uPA antibody. **B and D**. Total RNA was isolated from HeLa cells treated under similar conditions and cyclin D1 and uPA mRNA levels were detected by semi-quantitative RT-PCR. Actin was used as loading control for both western blot and RT-PCR.

Our previous results have shown that TSA inhibits c-Jun expression and thereby it suppresses PMA-induced OPN transcription. To investigate whether TSA altered the expression of two well known AP-1 regulated genes such as cyclin D1 and uPA, HeLa cells were pretreated with TSA and then with PMA and levels of cyclin D1 and uPA were analyzed by western blot and reverse transcription-PCR. The data revealed that TSA suppressed PMA-induced expression of both cyclin D1 (Figure 6A and Figure 6B) and uPA (Figure 6C and Figure 6D) in dose dependent manner both at protein and mRNA levels respectively.

### TSA suppresses cervical tumor growth in response to PMA in NOD/SCID mice xenograft model

**Figure 7:**
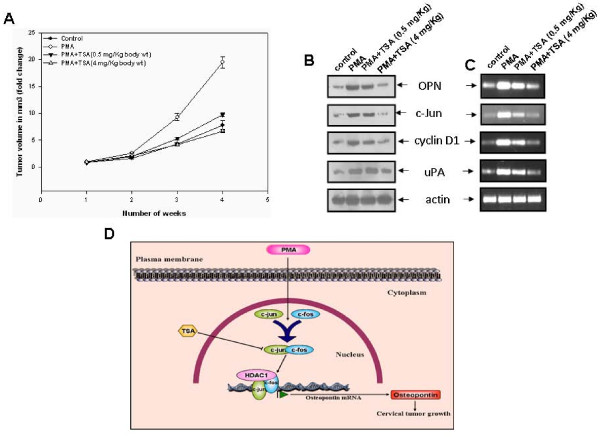
**TSA suppresses cervical tumor growth in response to PMA in NOD/SCID mice model**. **A**. HeLa cells (1 × 10^6^/0.2 ml) were mixed with matrigel (1:1 ratio) and injected subcutaneously into the flanks of mice. Six mice were kept in each group. Either PMA alone or in combination with two doses of TSA was injected intratumorally. After 4 weeks, mice were sacrificed and tumors were excised. The tumor volumes were calculated and the growth kinetics in tumor volume in fold change vs time in weeks is represented in the form of graph. The error bars represent the standard error of mean (SEM). *, p < 0.05. **B and C**. Tumor lysates were analyzed by western blot using anti-OPN, anti-c-Jun, anti-cyclin D1 and anti-uPA antibodies. Tumors were also analyzed by semiquantitative RT-PCR using gene specific primers. Actin was used as control. **D**. Schematic representation of PMA-induced OPN transcription through c-Jun leading to enhanced cervical tumor growth and TSA suppresses this process.

Our *in vitro *data further prompted us to extend this study to *in vivo *mice models. Therefore, to examine whether PMA promotes the cervical tumor growth and TSA plays any role in regulation of this process, HeLa cells were injected subcutaneously into the NOD/SCID mice. Either PMA alone or in combination with two doses of TSA was injected intratumorally. After 4 weeks, mice were sacrificed and tumors were excised. The tumor volumes were calculated and the growth kinetics of tumor volume in fold change vs time in weeks is represented in the form of graph (Figure 7A). The error bars represent the standard error of mean. The data revealed that PMA promoted tumor growth was significantly suppressed by TSA. The tumor lysates were analyzed for the expressions of OPN, c-Jun, cyclin D1 and uPA by western blot and RT-PCR (Figure 7B and Figure 7C). Higher expressions of these oncogenic proteins were observed in the lysates of tumors obtained from mice treated with PMA alone as compared to mice in the control group. These levels were substantially reduced in the lysates of tumors obtained from mice treated with two doses of TSA along with PMA (Figure 7B and Figure 7C). The typical tumor photographs are shown in Additional file 4, Figure S4A. The western blot and RT-PCR data were further confirmed by immunofluorescence analysis. The tumor samples were analyzed by immunofluorescence using anti-OPN, anti-c-Jun, anti-cyclin D1 and anti-uPA antibodies. The data revealed that the expression of these molecules was higher in tumors generated by PMA and TSA suppresses their expressions (Additional file 4, Figure S4B). This data demonstrates that OPN, c-Jun, cyclin D1 and uPA play crucial role in PMA-induced cervical tumor growth in NOD/SCID mice while TSA exerts its anti-tumor effects by downregulation of these oncogenic molecules.

## Discussion

HDAC inhibitors have emerged as promising chemotherapeutic agents. Previous studies have demonstrated that these inhibitors exhibit a range of anti-tumor activities including cancer cell cycle arrest, stimulation of differentiation and induction of apoptosis [35]. However, the mechanisms by which HDAC inhibitors show their anticancer activities are not completely understood and remain to be elucidated [44]. Earlier reports have shown that these inhibitors can have effect on gene expression, induce accumulation of acetylated histones and enhance protein degradation, all of which can contribute to the anti-proliferative effects [33,45,46]. A possible mechanism by which these inhibitors control cancer cell growth is through downregulation of expression of proteins involved in proliferation and metastasis. One such protein is OPN, elevated levels of which have been linked to enhanced metastatic phenotype. To examine this hypothesis, we investigated the effect of TSA on PMA-induced OPN transcription. In this study, we observed that TSA suppresses OPN transcription in a dose dependent manner both at protein and mRNA levels.

Human OPN promoter deletion and mutagenesis analyses led to the characterization of AP-1 transcription factor being involved in regulation of OPN transcription in response to PMA. Its role in this phenomenon was further confirmed by performing electrophoretic mobility shift assay (EMSA). Supershift assay revealed the presence of c-jun/c-fos heterodimer at the OPN promoter. The *in vivo *recruitment of c-jun to the OPN promoter was established by chromatin immunoprecipitation assay. TSA was observed to inhibit this recruitment. Moreover, Sakata et al have demonstrated that TSA activates the OPN gene promoter through AP-1 site in mouse undifferentiated mesenchymal cell line [47]. We addressed the question how the recruitment of c-jun to the OPN promoter was affected by TSA in cervical cancer cells. One possible mechanism could be by changing the c-jun expression. To validate this hypothesis, c-jun expression was analyzed by western blot as well as by RT-PCR. Interestingly, the data revealed that TSA inhibited c-jun transcription affecting both protein and mRNA levels which leads to the reduction in c-jun binding to the OPN promoter. To our surprise, c-fos levels were unchanged by PMA and TSA treatment. We also investigated the acetylation status of histones H3 and H4 associated with the AP-1 binding sequence of OPN promoter. Employing ChIP assays, we observed that TSA inhibits the hyperacetylation of histones H3 and H4 which is induced by PMA. This could be one of the mechanisms by which OPN expression is suppressed by TSA since acetylation status of the histones is directly correlated with gene expression. This report also showed that TSA can block the recruitment of components of the preinitiation complex viz RNA polymerase II and TFIIB to the OPN promoter. These findings were consistent with previous evidence that HDAC activity is required to recruit the preinitiation complex to selected genes that are activated by the transcription factors Stat 1, Stat 2 and Stat 5 [48,49].

Histone deacetylases have been divided into different classes. HDAC1, but not HDAC3 and HDAC4 seemed to be required for OPN transcription in response to PMA as determined by siRNA transfection experiments. OPN promoter activity was significantly reduced upon silencing HDAC1 suggesting its importance in the regulation of OPN expression by PMA. Since it was observed that TSA inhibits the PMA-induced OPN transcription by altering the expression of c-jun, we sought to determine whether TSA has any effect in regulation of expression of other c-jun specific target genes. Indeed, the expression of two well-known AP-1 regulated genes, cyclin D1 and uPA, was suppressed by TSA suggesting the strong anti-tumor activities of TSA. HDAC inhibitors have more profound effect on the growth of tumor cells than the normal cells [50]. Tumor cells have increased levels of OPN, c-jun, cyclin D1 and uPA. Our results revealed that the expression levels of OPN, c-Jun and c-Fos are higher in SiHa as compared to HeLa cells. This might be due to the fact that SiHa cells are more invasive than HeLa cells. All these oncogenic molecules have been shown to play important role in regulation of tumor growth, proliferation and metastasis. Therefore, it may be hypothesized that the growth inhibitory activities of TSA might be due to the down regulation of OPN, c-jun, cyclin D1 and uPA in both HeLa and SiHa cells.

In summary, this is the first time we report that TSA suppresses the PMA-induced transcription of OPN in cervical cancer model. Thus, this evidence suggested that the transcription factor, AP-1 has the binding ability to the OPN regulatory region that ultimately regulates the expression of OPN gene, which leads to tumor progression in cervical cancer (Figure 7D). Thus, inhibiting OPN expression at the transcriptional level by TSA might provide a novel strategy for the prevention of cervical cancer.

## Conclusions

This study has investigated the effects of HDAC inhibitor, TSA on OPN transcription and characterized the transcription factor binding site in the OPN promoter that plays an important role in this phenomenon. The data revealed that TSA suppresses the PMA-induced OPN gene expression in a dose dependent manner and induction of OPN transcription by PMA is mediated via AP-1 transcription factor that forms a heterodimer of c-jun and c-fos at the OPN promoter. The results also indicated that TSA suppressed the PMA-induced c-Jun recruitment to the OPN promoter by inhibiting c-Jun expression. The PMA-induced hyperacetylation of histones H3 and H4 associated with OPN promoter is also inhibited by TSA. Moreover, PMA promoted cervical tumor growth was significantly reduced by TSA in mice xenograft model. All these results highlight the mechanism of action of TSA and suggest that the growth inhibitory and anti-tumor activities of TSA might be exhibited, in part, by downregulation of OPN expression. Furthermore, this study represents OPN as possible candidate for anticancer therapies and blocking OPN expression at transcriptional level by TSA might provide a new strategy for management of cervical cancer.

## Competing interests

The authors declare that they have no competing interests.

## Authors' contributions

PS designed and performed most of the experiments and initially drafted the manuscript. SK carried out reverse-transcription PCR, helped the animal experiments and performed the statistical analysis. GCK contributed to the conception and final editing of the manuscript. All authors read and approved the final manuscript.

## Supplementary Material

Additional file 1 **TSA inhibits OPN transcription in SiHa cells**. A. SiHa cells were treated with 0-1 μM TSA for 6 h. Whole cell lysates were analyzed by western blot using anti-OPN antibody. B. SiHa cells were incubated with TSA under similar conditions as described above. Total RNA was isolated and the levels of OPN mRNA were detected by semiquantitative RT-PCR and analyzed by agarose gel electrophoresis. Actin was used as control. Click here for file

Additional file 2 **Schematic representation of a proximal segment of human OPN promoter and its sequence showing various transcription factor binding sites**. Click here for file

Additional file 3 **TSA suppresses c-Jun but not c-Fos expression in SiHa cells**. A and C. SiHa cells were treated with TSA (0-2 μM) for 3 h. Cell lysates (50 μg) containing equal amount of total proteins were analyzed by western blot using either anti-c-Jun or anti-c-Fos antibody. B and D. SiHa cells were incubated with TSA under similar conditions as described above. Total RNA was isolated and the levels of c-jun and c-fos mRNAs were detected by semiquantitative RT-PCR. Actin was used as control. Click here for file

Additional file 4 **TSA suppresses PMA-promoted cervical tumor growth in mice xenograft model**. A. The tumors were generated by injecting HeLa cells subcutaneously into the flanks of female NOD/SCID mice. Either PMA alone or in combination with two doses of TSA was injected intratumorally. The tumors were excised and typical tumor photographs are shown. B. Tumor samples were analyzed by histopathology and immunofluorescence using anti-OPN, anti-c-Jun, anti-cyclin D1 and anti-uPA antibodies and stained with Cy3-conjugated IgG. Click here for file
