# Supplementary figures and images for: Transcriptional regulation of human osteopontin promoter by histone deacetylase inhibitor, trichostatin A in cervical cancer cells
Source: Mol Cancer. 2010 Jul 7;9:178. doi: 10.1186/1476-4598-9-178 (PMC2911447; doi:10.1186/1476-4598-9-178)

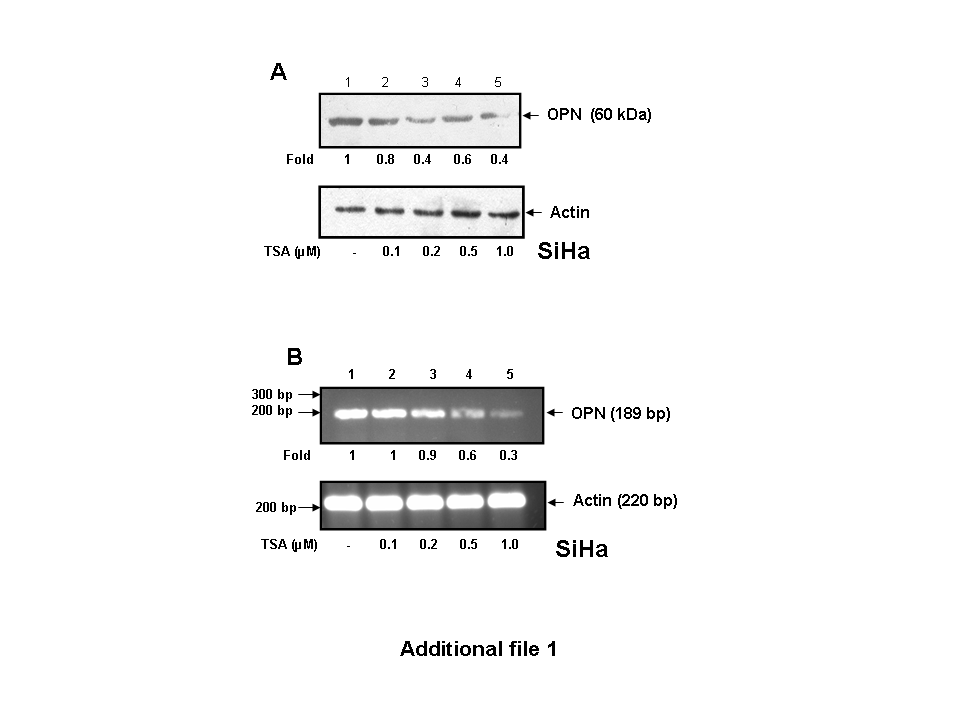

Supplement: Additional file 1 — TSA inhibits OPN transcription in SiHa cells. A. SiHa cells were treated with 0-1 μM TSA for 6 h. Whole cell lysates were analyzed by western blot using anti-OPN antibody. B. SiHa cells were incubated with TSA under similar conditions as described above. Total RNA was isolated and the levels of OPN mRNA were detected by semiquantitative RT-PCR and analyzed by agarose gel electrophoresis. Actin was used as control. [file 1476-4598-9-178-S1.TIFF]

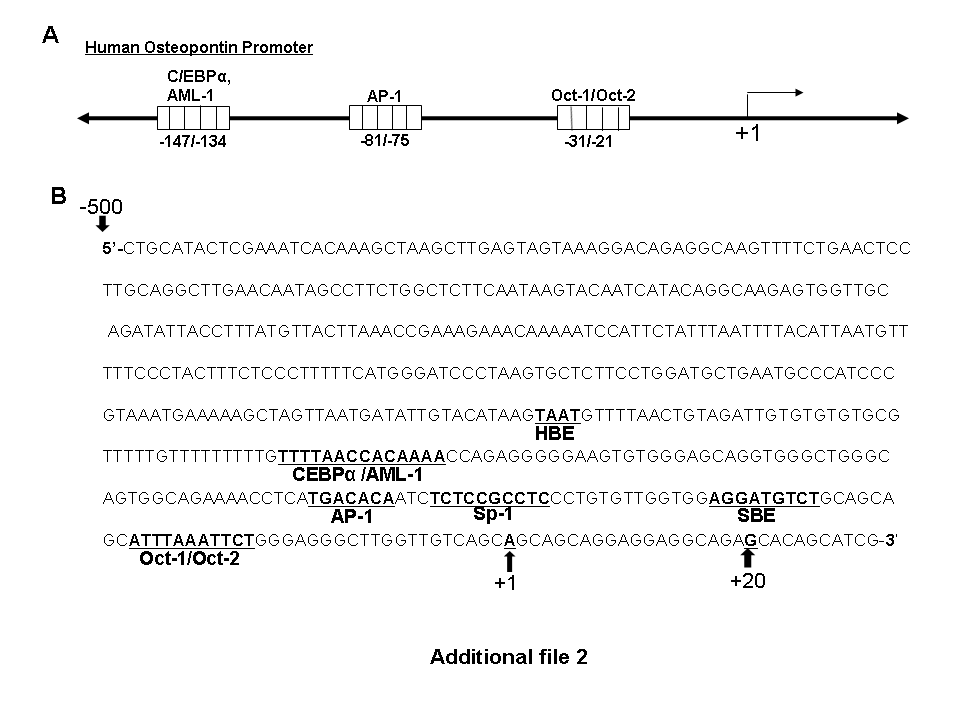

Supplement: Additional file 2 — Schematic representation of a proximal segment of human OPN promoter and its sequence showing various transcription factor binding sites. [file 1476-4598-9-178-S2.TIFF]

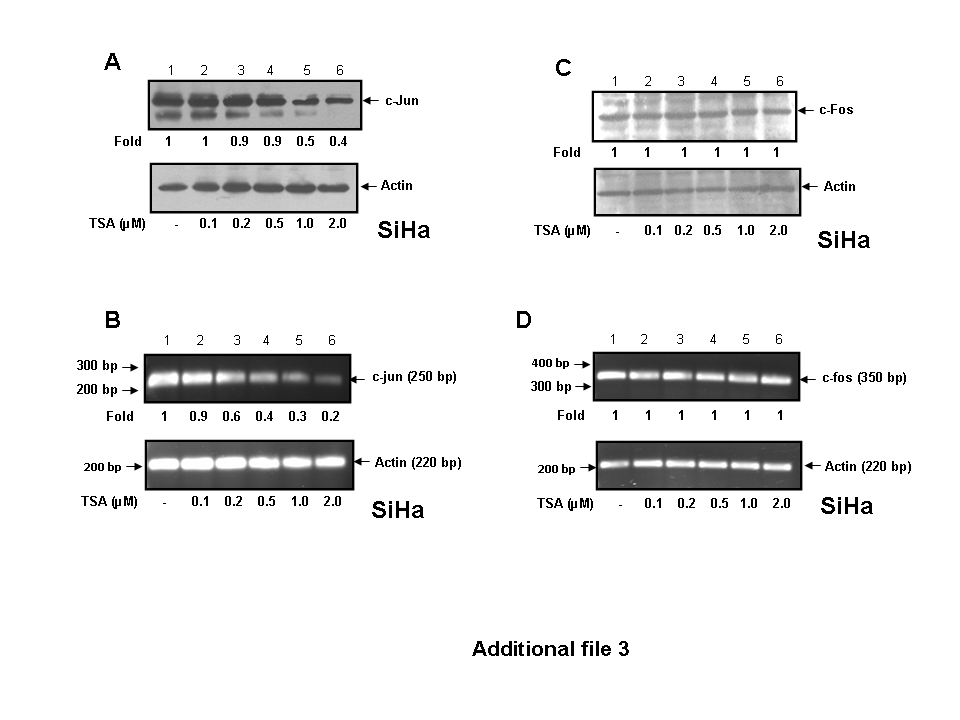

Supplement: Additional file 3 — TSA suppresses c-Jun but not c-Fos expression in SiHa cells. A and C. SiHa cells were treated with TSA (0-2 μM) for 3 h. Cell lysates (50 μg) containing equal amount of total proteins were analyzed by western blot using either anti-c-Jun or anti-c-Fos antibody. B and D. SiHa cells were incubated with TSA under similar conditions as described above. Total RNA was isolated and the levels of c-jun and c-fos mRNAs were detected by semiquantitative RT-PCR. Actin was used as control. [file 1476-4598-9-178-S3.TIFF]

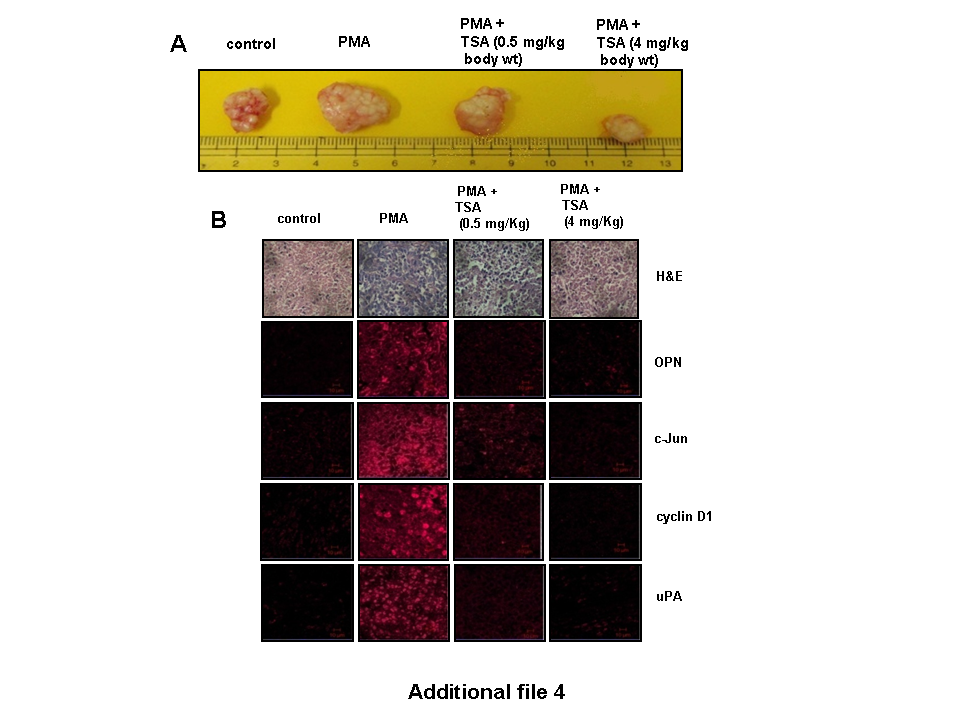

Supplement: Additional file 4 — TSA suppresses PMA-promoted cervical tumor growth in mice xenograft model. A. The tumors were generated by injecting HeLa cells subcutaneously into the flanks of female NOD/SCID mice. Either PMA alone or in combination with two doses of TSA was injected intratumorally. The tumors were excised and typical tumor photographs are shown. B. Tumor samples were analyzed by histopathology and immunofluorescence using anti-OPN, anti-c-Jun, anti-cyclin D1 and anti-uPA antibodies and stained with Cy3-conjugated IgG. [file 1476-4598-9-178-S4.TIFF]
